# The role of frailty in predicting mortality and readmission in older adults in acute care wards: a prospective study

**DOI:** 10.1038/s41598-018-38072-7

**Published:** 2019-02-04

**Authors:** Qiukui Hao, Lixing Zhou, Biao Dong, Ming Yang, Birong Dong, Yuquan Weil

**Affiliations:** 1 The Center of Gerontology and Geriatrics/National Clinical Research Center for Geriatrics, West China Hospital, Sichuan University, Chengdu, China; 2 Key Laboratory of Biotherapy and Cancer Center/Collaborative Innovation Center for Biotherapy, West China Hospital, Sichuan University, Chengdu, China

## Abstract

Few studies have focused on frailty as a predictor of mortality and readmission among inpatients in the acute care setting, especially over long follow-up periods. We conducted this study to determine the impact of the frailty on subsequent mortality and readmission in this setting. This study was a prospective observational study conducted in the acute geriatric wards, with a three-year follow-up duration. We assessed frailty via the 36-item Frailty Index (FI), and a cut-off value of 0.25 was used to identify the presence or absence of frailty. We collected survival and readmission information through telephone interviews at 12, 24, and 36 months. We used the Cox regression model to examine the association between frailty and outcomes interested (death and readmission). The present study included 271 patients (mean age: 81.1 years old; 20.3% females), of whom 21.4% died during the 3-year follow-up period. One hundred and thirty-three patients (49.1%) were identified as being frail. The prevalence of frailty was similar in men and women (46.8% vs.58.2%, *P* = 0.130). Compared with non-frail patients, death and hospital readmission rates of frail patients were increased. Frailty was an independent predictor of 3-year death (adjusted hazard ratio (HR): 2.09; 95% confidence interval (CI): 1.20 to 3.63) and readmission (adjusted HR: 1.40; 95% CI: 1.04 to 1.88) after adjusting for several potential confounders. Frailty is prevalent among older inpatients and is a valuable predictor of 3-year mortality and hospital readmission in an acute care setting.

## Introduction

Frailty is defined as a geriatric syndrome or state, which is marked by increased vulnerability to even small stressors and decreased physiological reserve in old people^1–3^. The prevalence of frailty varies from 4.0–59.1% among community-dwelling older individuals^4^ and 19.0–75.6% nursing homes^5^, with high estimate variability stemming from differences between the assessment tools and the characteristics of populations in a given study. Previous studies that have specifically focused on the prevalence of frailty in acute care settings have estimated it to affect 33.5–68.5% (median) of older patients in this context^6–8^.

Numerous studies have conclusively demonstrated that frailty is a predictor for many adverse health outcomes, including disability, falls, delirium, hospitalization, and mortality up to 12 years follow up period^6,9–14^. However, the majority of these previous studies have focused on the association between frailty and mortality among community-dwelling older persons^9–11^ or residents in long-term care settings^12,15^, rather than on those in acute care settings.

Studies regarding the role of frailty in predicting adverse events in acute care settings have begun to emerge, as they can help formulate patient care plans and improve shared decision making regardless of how frailty is defined^8,16–20^. Of these studies, the most commonly used frailty assessment tools were the Frailty Index, the Clinical Frailty Scale, and the Fried Frailty Phenotype^8^. Recently, a large cohort study developed a hospital frailty risk score based on the International Statistical Classification of Diseases and Related Health Problems, Tenth Revision (ICD-10) diagnostic codes and found frailty was significantly associated with 30-day mortality (odds ratio: 1.71, 95% CI: 1.68 to 1.75) and 30-day readmission (1.48, 1.46 to 1.50)^21^. This hospital frailty risk score showed moderate agreement with the Frailty Index (Pearson’s correlation coefficient 0.41). These studies have thus demonstrated that frailty is an important prognostic factor when it comes to predicting adverse health outcomes in older patients in acute care settings. Notably, follow-up periods in these past studies have varied widely from 30 days to 12 months.

The value of frailty as a predictor longer-term adverse events has to date remained unclear in acute care settings. Furthermore, there are still some contradictory results with regard to how well frailty in predictive of readmission in such settings^22–25^ and data from non-Caucasian inpatient populations are scarce. Therefore, we designed this study to examine the prevalence of frailty as defined by frailty index in a Chinse patients of older inpatients in an acute care setting and to explore the value of frailty as a predictor of mortality and readmission for this population over a long-term (3-year) follow-up period.

## Methods

### Study design and patients

This study was a prospective observational study conducted in the Center of Gerontology and Geriatrics, West China Hospital, Sichuan University. All patients who were admitted to the ward in 2012 and provided formal consent were considered for inclusion in our study. We excluded those patients with severe disease because they did not complete the questionnaire or functional assessments required for this study. Trained volunteers (medical students) collected health-related variables using a pre-designed general questionnaire through face-to-face interviews within 48 hours of admission. We assessed the health-related and functional variables based on the status at admission. Before data collection, research staff trained all volunteers using investigation manuals, videos, and simulated patients. All volunteers passed a training test before participating in the formal investigation. We performed all methods in accordance with the relevant guidelines and regulations in the present study.

### Frailty assessment

In the present study, we constructed a frailty index (FI) using 36 health-related deficits/variables, according to a standard procedure^26^. All considered variables consisted of acute and chronic diseases present as diagnosed by an attending physician within 48 hours of admission (n = 12), sarcopenia assessed by research team according to the guidelines of the modified Asia Working Group for Sarcopenia (32), 14 items in Activities of Daily Living (ADL) or Instrumental ADL (IADL) items, and 9 symptoms including psychological and memory complaints. For binary variables, results were coded with a value of “1” indicating that the variable present and “0” indicating it was absent. For variables (ADL and IADL items) with 3 possible values, intermediate responses were coded as “0.5”. We did not include the BMI as a part of the frailty index in this study for the cutoff of BMI was inconsistent among Chinese people^27–29^. All items in the FI did not have missing data and were showed in Table 1. For each old individual, we calculated the FI as the sum of all score of present deficits divided by the total number of considered deficits (n = 36). Theoretically, the FI would be ranging between 0 and 1 as a continuous score. We chose 0.25 as cut-off point of the FI according to previous studies^12,30–34^.

**Table 1:** Health-related deficits/variables used to construct the Frailty index.

	Health-related deficits/variables	Recode
1	Hypertension	No = 0, yes = 1
2	Coronary heart disease	No = 0, yes = 1
3	Diabetes	No = 0, yes = 1
4	Chronic obstructive pulmonary disease	No = 0, yes = 1
5	Gastrointestinal disease	No = 0, yes = 1
6	Liver disease	No = 0, yes = 1
7	Kidney disease	No = 0, yes = 1
8	Stroke	No = 0, yes = 1
9	Osteoarthrosis	No = 0, yes = 1
10	Cancer	No = 0, yes = 1
11	Cataract	No = 0, yes = 1
12	Deafness	No = 0, yes = 1
13	Chest tightness or chest pain	No = 0, yes = 1
14	Dizziness	No = 0, yes = 1
15	Obvious memory loss	No = 0, yes = 1
16	Transient speechless or aphasia	No = 0, yes = 1
17	Joint pain	No = 0, yes = 1
18	Cough	No = 0, yes = 1
19	Falls	No = 0, yes = 1
20	Fracture	No = 0, yes = 1
21	Shortness of breath or edema	No = 0, yes = 1
22	Sarcopenia	No = 0, yes = 1
23	Eating	Independent = 0; need some help = 0.5; depended = 1
24	Grooming	Independent = 0; need some help = 0.5; depended = 1
25	Dressing	Independent = 0; need some help = 0.5; depended = 1
26	Going to bed	Independent = 0; need some help = 0.5; depended = 1
27	Bathing or showering	Independent = 0; need some help = 0.5; depended = 1
28	Indoor activities	Independent = 0; need some help = 0.5; depended = 1
29	Using the toilet	Independent = 0; need some help = 0.5; depended = 1
30	Cooking	Independent = 0; need some help = 0.5; depended = 1
31	Finance management	Independent = 0; need some help = 0.5; depended = 1
32	Take public vehicles	Independent = 0; need some help = 0.5; depended = 1
33	Shopping	Independent = 0; need some help = 0.5; depended = 1
34	Walking about 200 meters	Independent = 0; need some help = 0.5; depended = 1
35	Cutting nails	Independent = 0; need some help = 0.5; depended = 1
36	Using stairs to up and down one floor	Independent = 0; need some help = 0.5; depended = 1

### Mortality and readmission information

We obtained mortality and readmission information for all patients through telephone interviews conducted at 12, 24, and 36 months from the baseline by trained research volunteers. We also confirmed the survival information based on records in the Death Registry of Sichuan province with the approval of local government. We recorded the death dates of patients who died during the follow-up period. For those patients who did not die during the follow-up period, we recorded the date of the last follow-up. For patients who were readmitted to the hospital more than one time during follow-up, we record the first date of readmission.

### Co-variables

To assess the influence of variables not encoded directly in the frailty index, we also collected the following variables from the medical records systems and face-to-face interviews for each patient: age, sex, body mass index (BMI) as calculated by the body weight (Kg) divided by the square of the body height (m), systolic and diastolic blood pressure (SBP and DBP), education levels (illiteracy, primary school, secondary school, or advanced), previous occupations (intellectual work, light physical labor, heavy physical labor), living status (living alone or not), marriage status (married and living with spouse or not), lifestyle factors (smoking and alcohol consumption status).

### Statistical analyses

All statistical analyses were performed using the SPSS Statistics for Windows, Version 17.0. Chicago: SPSS Inc., with P-values of <0.05 (two-tailed) indicating the presence of a statistically significant difference between the two groups. We presented the baseline characteristics of the patients by the frailty status (frailty and no frailty). We presented continuous variables and categorical variables as means ± standard deviations or numbers and proportions, respectively. We tested the differences between frailty and no frailty groups using independent Student’s t-tests or Chi-squared tests according to the types of variables. We used the Spearman correlation to assess the relationships between frailty index and age. We used the Cox regression models to estimate the hazard ratio (HR) and its 95% confidence interval (CI) of the frailty status as an independent variable in functions of increased risk of mortality and readmission. In adjusted model 1, we adjusted the general variables (age, sex, and education). In adjusted model 2, we adjusted several other potential confounders (*P* < 0.2, co-variables when compared among frailty and no frailty groups). We used the Kaplan–Meier method to plot the cumulative risk curves for death and readmission, and the differences between these curves were examined using the log-rank test.

### Ethics

Research Ethics Committee in Sichuan University approved the performance of the study. We also obtained written informed consent all patients or their legal proxies before the study began.

## Results

### The characteristics of the study patients

A total of 313 patients agreed to participate in this study. We excluded 25 subjects due to severe disease because they did not complete the questionnaire or functional assessments required for this study. A total of 17 patients were lost due to failure to follow-up. We included 271 inpatients (55, 20.3% females) in the present study. The age of the patients ranging from 60 to 101 (mean: 81.1 ± 6.6 years). The maximum of FI is 0.69. The mean (standard deviation) and median FI scores of the patients were 0.26 (0.16) and 0.22, respectively. The 99th percentile of FI in this sample was 0.68. The FI follows an approximately normal distribution (Skewness = 0.671, standard error = 0.148; Kurtosis = −0.208, standard error = 0.295) and was positively related to age (r = 0.26, *P* < 0.001). According to the FI cutoff (0.25), 133 (49.1%) patients were identified as being frail.

The percentage of frailty was similar in females and males (58.2% vs. 46.8%, X^2^ = 2.289, p = 0.130). Female had similar FI scores to those of male (0.28 ± 0.15 vs. 0.26 ± 0.16; t = −0.71, *P* = 0.479). Frail patients were older compared to non-frail patients (79.3 ± 7.6 vs. 82.8 ± 4.9; t = 4.48, p < 0.001). The frequency of married and live with spouse was lower in frail patients than in non-frail patients (67.7% vs. 79.7%, X^2^ = 5.08, *P* = 0.024). Table 2 shows the characteristics of the study patients by their frailty status (frailty or no frailty).

**Table 2:** Characteristics of the study patients according to frailty status.

	Total (n = 271)	No frailty (n = 138)	Frailty (n = 133)	P value
Age (years)	81.1 ± 6.6	79.3 ± 7.6	82.8 ± 4.9	<0.001**
Age group (80 + years old) (n, %)	178 (65.7)	81 (58.7)	97 (72.9)	0.014*
Female (n, %)	55 (20.3)	23 (16.7)	32 (24.1)	0.130
BMI (kg/m^2^)	22.5 ± 3.6	22.8 ± 3.5	22.1 ± 3.8	0.161
SBP (mmHg)	126.2 ± 14.5	125.7 ± 14.1	126.7 ± 14.8	0.546
DBP (mmHg)	71.5 ± 9.7	71.5 ± 9.1	71.6 ± 10.3	0.925
Frailty index	0.26 ± 0.16	0.13 ± 0.06	0.39 ± 0.12	<0.001**
**Education level**				
Illiteracy (n, %)	8 (3.0)	4 (2.9)	4 (3.0)	
Primary school (n, %)	41 (15.1)	18 (13.0)	23 (17.3)	
Secondary school or advanced (n, %)	222 (81.9)	116 (84.1)	106 (79.7)	0.616
**Previous occupations**				
Intellectual work (n, %)	225 (83.0)	112 (81.2)	113 (85.0)	
Light physical labor (n, %)	30 (11.1)	18 (13.0)	12 (9.0)	
Heavy physical labor (n, %)	16 (5.9)	8 (5.8)	8 (6.0)	0.573
Live alone (n, %)	31 (11.4)	15 (10.9)	16 (12.0)	0.764
**Marital status**				
Married and live with spouse (n, %)	200 (73.8)	112 (79.7)	88 (67.7)	0.024*
Current smokers (n, %)	26 (9.6)	16 (11.6)	10 (7.5)	0.255
Current alcohol drinkers (n, %)	31 (11.4)	20 (14.5)	11 (8.3)	0.108

Note: Data are the mean ± SD unless otherwise indicated. Abbreviations: BMI, body mass index; SBP, systolic blood pressure; DBP, diastolic blood pressure; SD, standard deviation. **P < 0.01; *P < 0.05.

### The relationship between frailty and all-cause mortality

**Figure 1:**
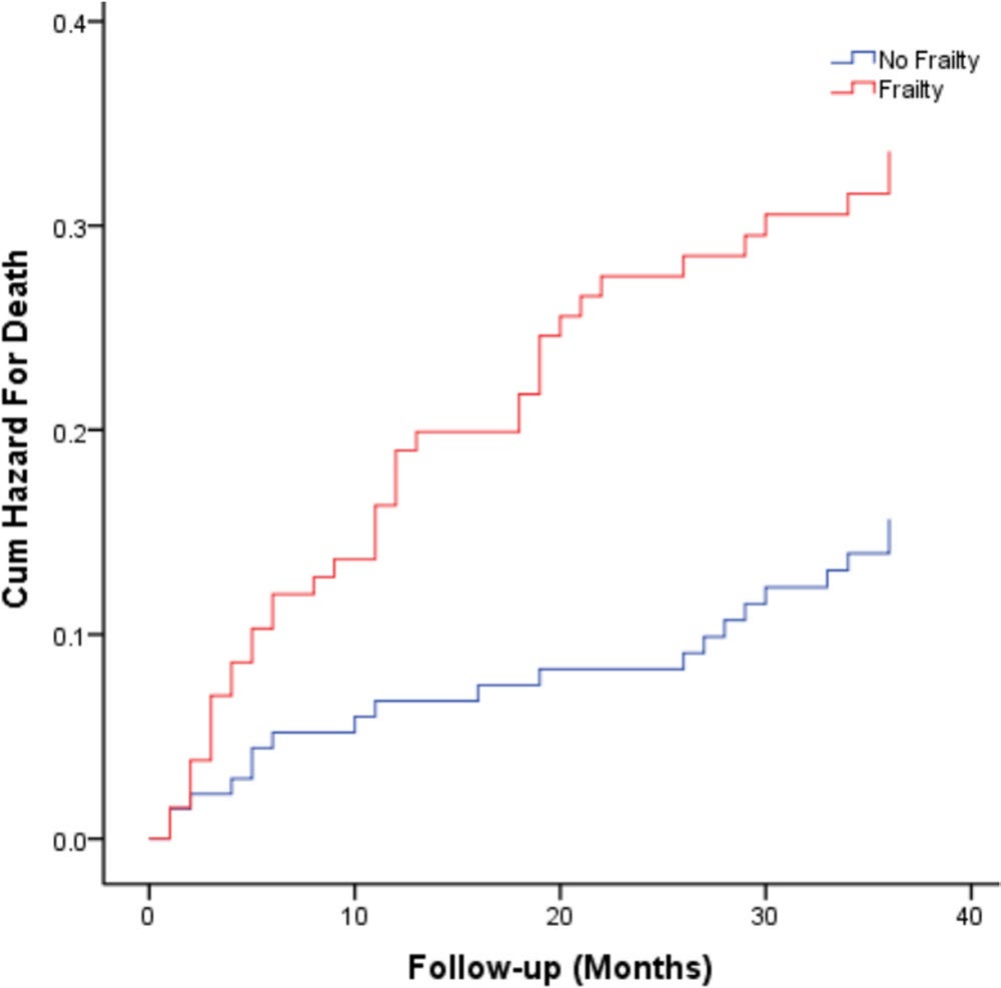
Cumulative risk curves of the study patients for death, according to frailty at baseline (the two curves significantly differs in the Log-rank test, p = 0.004).

Compared with non-frail patients, 3-year death rates of frail patients were significantly increased (28.6% vs. 14.5%, X^2^ = 7.98, *P* = 0.005). Table 3 shows the results of Cox regression models for frailty status, as a function of increased risk of death. Compared to the no frailty group, frail patients had a higher risk of death (hazard ratio: 2.18, 95% confidence interval (1.27 to 3.74), *P* = 0.005) than those who were not frail. After adjustment for age, sex, education levels, BMI, marital status, and alcohol intake, the model was stable and also found that frail subjects had a higher risk of death compared to the no frailty group (hazard ratio: 2.09, 95% confidence interval (1.20 to 3.63), *P* = 0.009). Figure 1 shows the cumulative risk curves for the death of the study patients by their frailty status at baseline, and these two curves differ significantly in the two groups (Log-rank *P* = 0.004).

**Table 3:** Association between frailty and adverse outcomes (3-year follow-up) according to Cox regression models.

	Mortality		Hospital readmission	
	No frailty	Frailty	No frailty	Frailty
**Unadjusted**				
HR (95% CI) P	Reference (1)	2.18 (1.27–3.74) 0.005	Reference (1)	1.45 (1.08–1.94) 0.013
Adjusted model 1*				
HR (95% CI) P	Reference (1)	2.17 (1.26–3.76) 0.006	Reference (1)	1.44 (1.07–1.93) 0.016
Adjusted model 2**				
HR (95% CI) P	Reference (1)	2.09 (1.20–3.63) 0.009	Reference (1)	1.40 (1.04–1.88) 0.026

Note: *Adjusted model 1: Age, sex, education.

**Adjusted model 2: Age, sex, education, BMI, body mass index, marital status, alcohol intake.

### The relationship between frailty and readmission

**Figure 2:**
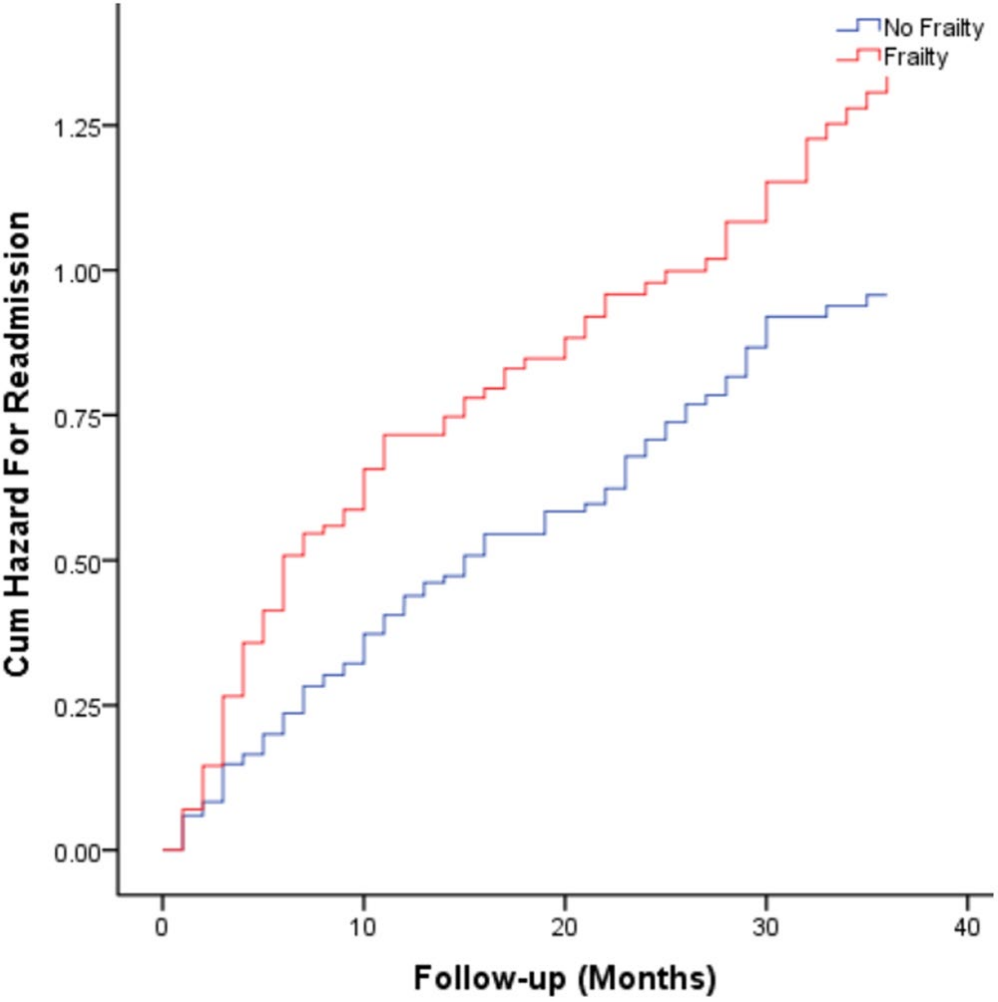
Cumulative risk curves of the study patients for readmission, according to frailty at baseline (the two curves significantly differs in the Log-rank test, p = 0.011).

Compared with non-frail patients, readmission rates of frail patients were significantly increased (73.7% vs. 61.6%, X^2^ = 4.51, *P* = 0.034). Table 3 shows the results of Cox regression models for frailty status, as a function of increased risk of readmission. Compared to the patients without frailty, frail patients had a higher risk of readmission (hazard ratio: 1.45, 95% confidence interval (1.08 to 1.94), *P* = 0.013) than non-frail patients. After adjustment for age, sex, education levels, BMI, marital status, and alcohol intake, the model was stable and also found that frail subjects had a higher risk of readmission as compared to the no frailty group (hazard ratio: 1.40, 95% confidence interval (1.04 to 1.88), *P* = 0.026). Figure 2 shows the cumulative risk curves for readmission of the study patients according to their frailty status at baseline, and these two curves differ significantly in the two groups (Log-rank *P* = 0.011).

## Discussion

In this study, we explored the prevalence of frailty and explored the relationship between frailty, mortality, and readmission in a population of older Chinse inpatients in an acute care setting. To the best of our knowledge, this is the first study to investigate the relationship between frailty, mortality, and readmission longitudinally in Chinese hospitalized older patients with a long-term (3-year) follow up duration. The present study showed that frailty was prevalent (49.1%) among older Chinese inpatients in acute geriatric wards. Frailty, as assessed by the Frailty Index, is a useful predictor of long-term death and readmission. This evidence indicates that we should consider frailty status when formulating health care plans for older inpatients.

A previous study with a large sample size (34,123 respondents) found that China has the lowest frailty prevalence than other countries (Ghana, India, Mexico, Russia and South Africa) among community-dwelling older persons^35,36^. The present study showed that the prevalence of frailty (49.1%) was similar to that identified by previous studies conducted in acute care settings in western countries (Europe, North America, Australia or New Zealand, 48.5%)^8^. Li and colleges previously used the FRAIL scale to define frailty and found that only 15.1% of patients with chronic diabetes in a geriatric ward met these criteria for frailty^37^. In our study, we chose a commonly used frailty index cut-off value (0.25) to define frailty in patients with acute conditions. This assessment methodology did influence the prevalence of frailty, potentially leading to a higher prevalence based on this definition for the FI captured more health-related information than FRAIL scale. The prevalence of frailty has varied massively in previous studies, ranging from 1.1–100% in studies conducted in western countries in acute care settings^8^. While it is difficult to compare frailty prevalence between studies due to variations in diagnostic methodology, our results suggest that unlike in community-dwelling older adults, older Chinese patients with acute illnesses have a similar prevalence of frailty as do those in other countries.

Some studies have suggested that frailty cannot be used as a predictor of readmission for those older patients with cirrhosis^23^ or idiopathic Parkinson’s disease^38^. In the two previous studies^23,38^, they recruited different patients and used different scales to define frailty with our present study. The reasons for these inconsistencies of the results were unclear and should be further investigated. Consistent with previous studies^6,7,20–22,24,25,39,40^, we found frailty to be a prognostic factor for mortality and readmission, independent of age, sex, and other potential confounders. Recently, Cesari and colleagues enrolled 4,488 patients aged 65 and older in 116 hospital wards in Italy between 2010 and 2016 and found that their 34-item frailty index was significantly predictive of 12-month mortality (HR 1.46, 95% CI 1.32–1.62) and in-hospital mortality after adjustment for age and sex^39^. Vidan and colleagues included 450 patients aged 79 and older and found frailty to be an independent predictor of 12-month readmission^40^. Our study extends these conclusions to a Chinese population and employs a more extended follow-up period (3 years).

There are certain limitations to our present study. First, we constructed our frailty index without using the same variables (70-item) employed in its original development study^41^. However, we constructed our frailty index according to a standard procedure and following the required criteria for variables selection^26^. Furthermore, the frailty index with 30 or more items have been shown to sufficiently capture frailty status and to be accurate for predicting adverse events, suggesting that our 36-item frailty index was sufficiently powerful^26^. We used 0.25 as the cut-off value for our frailty. This value has exhibited the strongest potential to predict adverse outcomes and is consistent with the results of the Fried Frailty phenotype and FRAIL scale^32–34^. Second, the number of patients in our study was smaller than many previous studies (n = 271), and we excluded patients with severe disease, which may have introduced a selection bias and thereby underestimated the prevalence of frailty. The small number of patients also limited our ability to perform subgroup analyses according to sex, different frailty levels and set more FI cut-off points. In addition, the small sample size may be the main reason for a wide 95% CI when we put FI in the Cox regression model as a continuous score. Third, we only included Chinese people (Han) in this study. Thus, we cannot extend the present conclusion to other ethnic groups or to other countries with medical care models distinct from those in China. Fourth, there were many pieces of missing data with regard to other potential confounders, such as income and physical activity levels, limiting our ability to adjust the model in light of these potential confounders. Additionally, the exposure of the studied patients to physiotherapy intervention during the ward stay as another possible confounder in the present study. Finally, the mean age of the patients in this study was 81 years. For such an old cohort, it is important to consider that a survival bias may be present when applying these findings.

## Conclusions

Frailty (as assessed by frailty index) is a prevalent state among older inpatients and is valuable as a predictor of 3-year mortality and hospital readmission in acute care settings. Given the limitations of our study, prospective studies with larger sample sizes using other frailty assessment methods and conducted in other acute care settings are warranted to confirm these findings.
